# Optimized feature selection and advanced machine learning for stroke risk prediction in revascularized coronary artery disease patients

**DOI:** 10.1186/s12911-025-03116-2

**Published:** 2025-07-24

**Authors:** Yong Si, Armin Abdollahi, Negin Ashrafi, Greg Placencia, Elham Pishgar, Kamiar Alaei, Maryam Pishgar

**Affiliations:** 1 University of Southern California, Los Angeles, CA USA; 2 California State Polytechnic University, Pomona, CA USA; 3 Colorectal Research Center, Iran University of Medical Sciences, Iran; 4 California State University, Long Beach, CA USA

**Keywords:** Revascularized CAD patients, Machine learning, Feature selection, Stroke risk prediction, CatBoost algorithm, SHAP analysis

## Abstract

**Background:**

Coronary artery disease (CAD) remains a leading cause of global mortality, with stroke constituting a significant complication among patients undergoing coronary revascularization procedures, such as percutaneous coronary intervention (PCI) or coronary artery bypass grafting (CABG). Previous research has demonstrated the successful application of machine learning (ML) in predicting various postoperative outcomes, including poor prognosis following cardiac surgery and the risk of postoperative stroke. Despite these advancements, a critical gap persists in studies quantitatively linking the risk of postoperative stroke to revascularization using ML-based approaches. This study aims to address this gap by developing and validating ML models to predict the risk of stroke in CAD patients undergoing coronary revascularization, with the ultimate goal of enhancing clinical decision-making and improving patient outcomes.

**Methods:**

We developed an ML framework to predict stroke risk in patients with CAD undergoing revascularization. A total of 5,757 patients were extracted from the Medical Information Mart for Intensive Care IV (MIMIC-IV) database. Feature selection was performed using a combination of Pearson correlation analysis, least absolute shrinkage and selection operator (LASSO), ridge regression, and elastic net. Initially, 35 features were identified based on expert opinion and a comprehensive literature review; the integrated results of the feature selection methods reduced the feature set to 14. The dataset was randomly divided into training, testing, and validation subsets with proportions of 70%, 15%, and 15%, respectively. Several ML models were evaluated, including logistic regression, XGBoost, random forest, AdaBoost, Bernoulli naive Bayes, k-nearest neighbors (KNN), and CatBoost. Model performance was assessed using the area under the receiver operating characteristic curve (AUC-ROC), accuracy, and 500 bootstrapped 95% confidence intervals (CIs) to ensure robust evaluation.

**Results:**

The CatBoost model demonstrated superior performance, achieving an AUC of 0.8486 (95% CI: 0.8124–0.8797) on the test set and 0.8511 (95% CI: 0.8203–0.8793) on the validation set. Shapley Additive Explanations (SHAP) analysis identified the Charlson Comorbidity Index (CCI), length of stay (LOS), and treatment types as the most influential predictors. Notably, compared to the best existing literature, which reported an AUC of 0.760 on the test set, our model exhibited a 9% improvement in predictive performance while utilizing a more parsimonious feature set.

**Conclusion:**

By integrating four feature selection methods, we significantly streamlined the feature set, resulting in a more efficient and reliable predictive model. We propose the CatBoost model for the prediction of postoperative stroke in patients with CAD undergoing coronary revascularization. With its high accuracy, the proposed model offers valuable insights for medical practitioners, enabling informed decision-making and the implementation of preventive measures to mitigate stroke risk.

## Introduction

Coronary Artery Disease, abbreviated as CAD, is characterized by the narrowing or blockage of the coronary arteries due to atherosclerosis, and it significantly contributes to the high mortality rates associated with cardiovascular diseases [1]. In 2022, heart disease remained the leading cause of death in the U.S., with 702,880 fatalities, equating to one in every five deaths. Coronary heart disease specifically was responsible for 371,506 deaths that year [2]. Revascularization procedures such as PCI and CABG are common and effective treatments for managing CAD [3]. While these revascularization procedures are endorsed by reducing symptoms of CAD, they have limitations and carry unignorable risks. A study from Finland reported that patients with a history of stroke were less than half as likely to receive PCI. Similarly, the presence of Dementia or Alzheimer’s disease reduced PCI use to the same degree as being over 85 years old [4, 5]. CABG also brings new problems. For instance, another study found that within 10 to 15 years after CABG, up to 40% of patients may require redo CABG due to recurrent angina, late myocardial infarction, and the need for additional intervention, leading to increased risk and cost [6, 7]. Stroke is also a significant risk factor following revascularization. Patients who suffered a stroke within 30 days of the procedure had a substantially higher five-year mortality rate [8–11]. The risk of stroke within the first year after revascularization was five times higher than in the age- and sex-matched general population. This risk was particularly elevated in patients with a history of stroke, diabetes mellitus, advanced age, male sex, and low socioeconomic status [12, 13]. The prediction of such adverse postoperative events is crucial for improving clinical decision-making and patient care.

Traditional risk models for perioperative stroke in coronary revascularization have historically relied on logistic regression and clinical risk scores. Recent studies using these conventional methods have identified consistent predictors of postoperative stroke, such as advanced age, prior cerebrovascular events, peripheral arterial disease, and emergent surgery. For example, a logistic-regression risk score developed for cardiac surgery patients (including CABG) found older age, history of stroke, peripheral occlusive disease, and prolonged cardiopulmonary bypass time to be independent predictors of stroke, albeit with modest discrimination (c-statistic 0.70) [14]. Similarly, analyses of large PCI cohorts using traditional multivariable models emphasize that patients with risk factors like carotid artery disease, atrial fibrillation, or hemodynamic instability (e.g. cardiogenic shock) are at significantly higher risk of an acute ischemic stroke after PCI [15]. While these clinical scoring systems help stratify risk, their predictive accuracy is limited by linear assumptions and predefined interactions.

Predicting postoperative stroke risk in revascularized CAD patients is crucial for improving clinical decision-making and patient outcomes. Recent studies have demonstrated the potential of machine learning models in predicting in-hospital mortality for ICU patients with heart failure, highlighting the impact of advanced feature selection and model optimization on predictive accuracy [16, 17]. However, research specifically linking stroke risk to revascularization procedures using ML remains limited. Existing studies often face challenges such as overfitting, inadequate feature selection, and class imbalances [18–21].

This research focuses on optimizing feature selection and enhancing ML model performance to predict postoperative stroke risk in revascularized CAD patients. We integrate traditional statistical methods with modern ML-based approaches to achieve both accuracy and explainability. Four robust statistical techniques – Pearson correlation, LASSO, Ridge, and Elastic Net regression – were employed to whittle down 35 candidate variables to 14 high-value predictors. These traditional methods assume mostly linear relationships, which makes them interpretable and stable in their selection (Elastic Net, for instance, balances sparse selection with stability in the presence of correlated features) [22]. However, purely linear methods can miss complex non-linear risk factors, as their predictive power is limited by linear assumptions. To address this, we complement the statistical filtering with machine learning-based feature importance analysis. Tree-based models like CatBoost and XGBoost can capture intricate patterns and interactions beyond pre-defined linear effects. By employing SHAP (Shapley Additive Explanations) on our CatBoost model, we gained nuanced insights into each feature’s contribution to stroke risk, both globally and for individual predictions [23]. This combined strategy retained only the most statistically and clinically relevant features, minimizing redundancy and complexity while preserving the ability to detect non-linear influences on outcomes. This aligns with broader trends toward efficient and interpretable modeling frameworks in machine learning [24, 25]. The streamlined 14-feature set improved model efficiency without sacrificing accuracy, and the use of SHAP enhanced interpretability, bridging the gap between a complex ML model and clinical understanding of risk factors [26]. Overall, our rigorous feature selection process mitigates overfitting and underscores the value of blending high-impact predictor selection via classical methods with advanced ML interpretability techniques for developing robust, generalizable clinical prediction models.

The proposed model demonstrated exceptional predictive performance, with the CatBoost algorithm achieving an AUC of 0.8486 (95% CI: 0.8124–0.8797) on the test set and 0.8511 (95% CI: 0.8203–0.8793) on the validation set. These results surpass those reported in prior studies, highlighting the effectiveness of the feature selection and preprocessing strategies employed. Consistent and high-performance metrics across datasets establish the CatBoost model as a reliable tool for predicting postoperative stroke risk in CAD patients.

The research underscores the pivotal role of data preprocessing in enhancing model accuracy and reliability. Missing values were addressed through random forest interpolation, maintaining data integrity for both categorical and numerical variables. To address the class imbalance between stroke and non-stroke cases, the study applied the Synthetic Minority Over-sampling Technique (SMOTE), which improved the model’s sensitivity to rare events. Prior work has shown that SMOTE can effectively enhance model performance in clinical settings by generating synthetic minority class samples and reducing bias in predictive analytics [27]. Additionally, categorical features were optimized through feature category reduction, mitigating overfitting and improving computational efficiency. These preprocessing steps collectively contributed to the model’s robustness and ensured its applicability in diverse clinical scenarios. This reflects a broader interest in training-efficient inference pipelines for time-series and decision-focused prediction [28]. Comparable efforts have emphasized the importance of realism and reproducibility in clinically-oriented datasets [29].

To improve model interpretability, SHAP analysis was incorporated, elucidating the contribution of individual predictors to the CatBoost model. The CCI emerged as the most significant variable, followed by LOS and treatment type. By visualizing the impact of these predictors, the study enhances the model’s transparency, making it a valuable decision-support tool for clinicians. Such interpretability plays a vital role in supporting confidence and appropriate reliance on AI-assisted tools in clinical contexts [30]. The integration of SHAP analysis bridges the gap between computational outputs and clinical applicability, ensuring predictions are both interpretable and actionable.

The study also emphasizes the importance of generalizability across diverse clinical settings. Prior research on mortality prediction in CAD patients has demonstrated the value of integrating machine learning models with clinical decision support systems to enhance patient risk assessment [31]. While SMOTE effectively balanced the dataset, we acknowledge its potential to alter class proportions and advocate for further validation using external datasets. This focus on generalizability reflects a commitment to developing predictive tools that are not only accurate but also practical for real-world implementation. Complementary efforts using sensor-driven clinical modeling in early-phase studies support the broader adoption of data-centric diagnostic tools [32]. Other recent studies have also emphasized the value of such data-driven modeling frameworks in clinical contexts [33] Recent advancements in language models have also underscored the potential of data-driven techniques in supporting healthcare delivery [34]. Collectively, these contributions advance the field of predictive analytics, offering a comprehensive framework for risk stratification and personalized care in CAD management.

## Methodology

### Data source

The MIMIC-IV database is a publicly accessible, comprehensive dataset that provides detailed health-related information on patients admitted to critical care units at the Beth Israel Deaconess Medical Center in Boston, Massachusetts [35]. Building upon its predecessor, MIMIC-III, MIMIC-IV offers an extensive collection of data, including patient admissions, demographics, vital signs, laboratory results, procedures, medications, caregiver notes, imaging reports, and detailed mortality information, such as dates and times. This robust dataset is specifically designed to support research in critical care, facilitating the development and validation of predictive models and enabling clinical studies aimed at enhancing patient outcomes. The breadth and depth of MIMIC-IVs records establish it as an invaluable resource for researchers investigating various dimensions of intensive care medicine.

### Data extraction

**Fig. 1:**
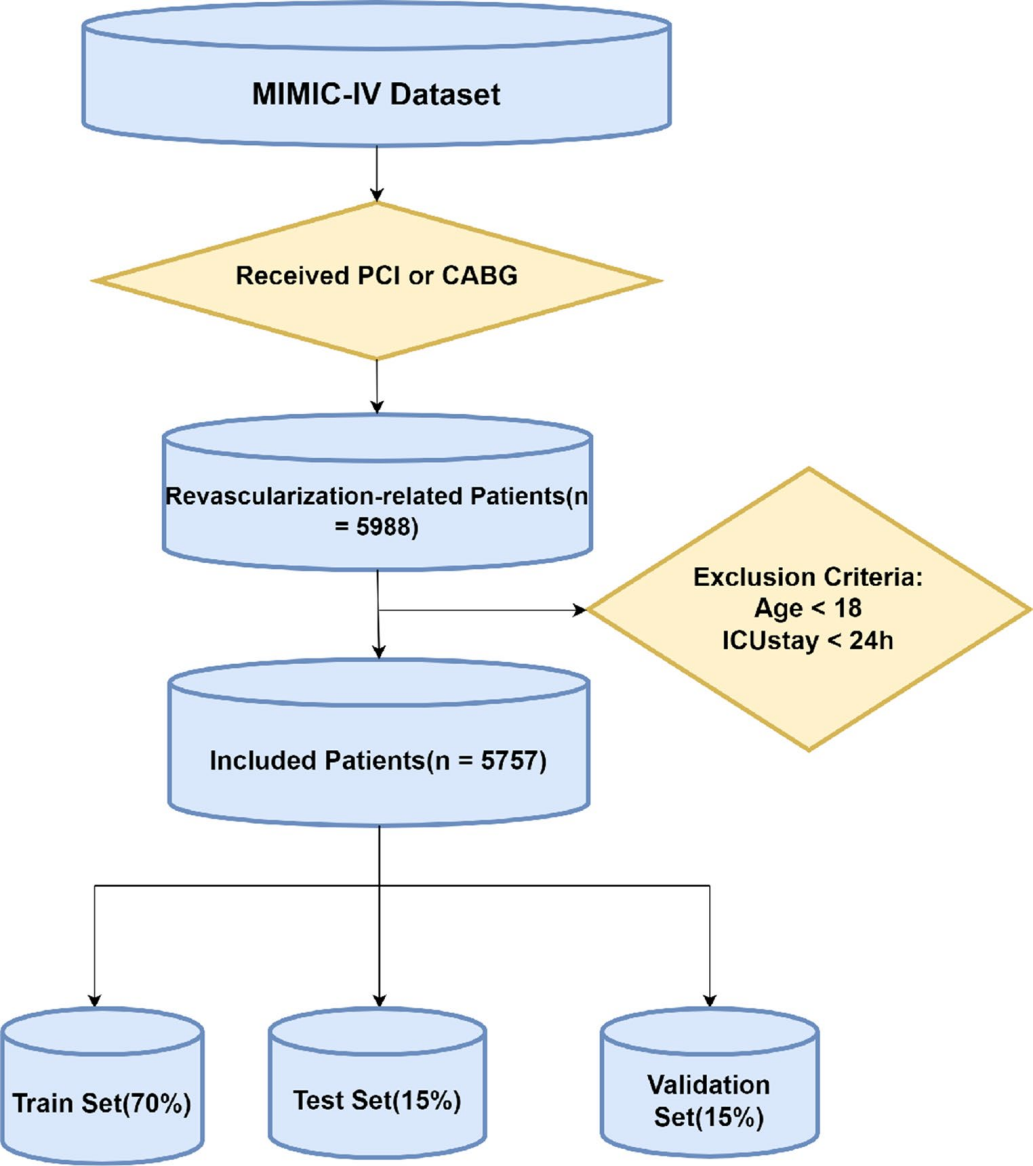
Flowchart of the data extraction procedure

Our study focused on adult intensive care unit (ICU) patients diagnosed with CAD who underwent revascularization procedures. Using text matching of ICD-9 and ICD-10 codes, we initially identified a cohort of 5,988 patients who had undergone either PCI or CABG. Patients under 18 years of age or with ICU stays of less than 24 hours were excluded, resulting in a final cohort of 5,757 adult patients with common clinical presentations. This refined dataset was designed to provide meaningful insights into the management and outcomes of CAD patients undergoing revascularization within critical care settings. The data extraction process is outlined in Fig. 1.

### Feature extraction and feature selection

Through a literature review and expert consultation, we identified and selected key features for analysis following data extraction. From a cohort of 5,757 patients, a comprehensive set of 35 features was curated, encompassing demographic, clinical, physiological, and treatment-related variables. Demographic variables included age, gender, race, insurance type, marital status, and the initial care unit (categorized as Cardiac Care Unit [CCU], Cardiovascular Intensive Care Unit [CVICU], or others). Clinical and physiological variables comprised the Charlson Comorbidity Index (CCI), Glasgow Coma Scale (GCS) score, and vital signs, including, systolic and diastolic blood pressure, temperature, heart rate, respiratory rate, and oxygen saturation. Additionally, a broad range of laboratory parameters were considered, such as hemoglobin, red blood cell distribution width, white blood cell count, platelet count, hematocrit, creatinine, prothrombin time (PT), international normalized ratio (INR), partial thromboplastin time (PTT), blood urea nitrogen (BUN), glucose, calcium, sodium, chloride, bicarbonate, and lactate levels. LOS was also included, serving as a surrogate marker for hospitalization complexity, postoperative recovery trajectory, and accumulated complication burden. While LOS is not a preoperative predictor, it was included for retrospective modeling to capture hospitalization complexity and adverse trajectories. Understanding the relationship between prolonged hospitalization and adverse events like stroke can help inform future model development, where proxy early indicators (e.g., early postoperative vitals, lab trends) could be used to approximate this risk in real-time. Thus, while LOS improves model performance retrospectively, it also highlights the importance of dynamic in-hospital predictors for prospective clinical deployment.

Treatment-related variables included revascularization modalities (CABG and PCI), history of stroke, antiplatelet usage, and LOS. The primary outcome, postoperative stroke, was defined as a binary variable (Yes/No). Pre-existing features were directly extracted from the MIMIC-IV database, while additional variables were derived using ICD codes and item IDs. This robust feature set was designed to ensure a comprehensive evaluation of factors influencing postoperative stroke outcomes in this patient population.

**Fig. 2:**
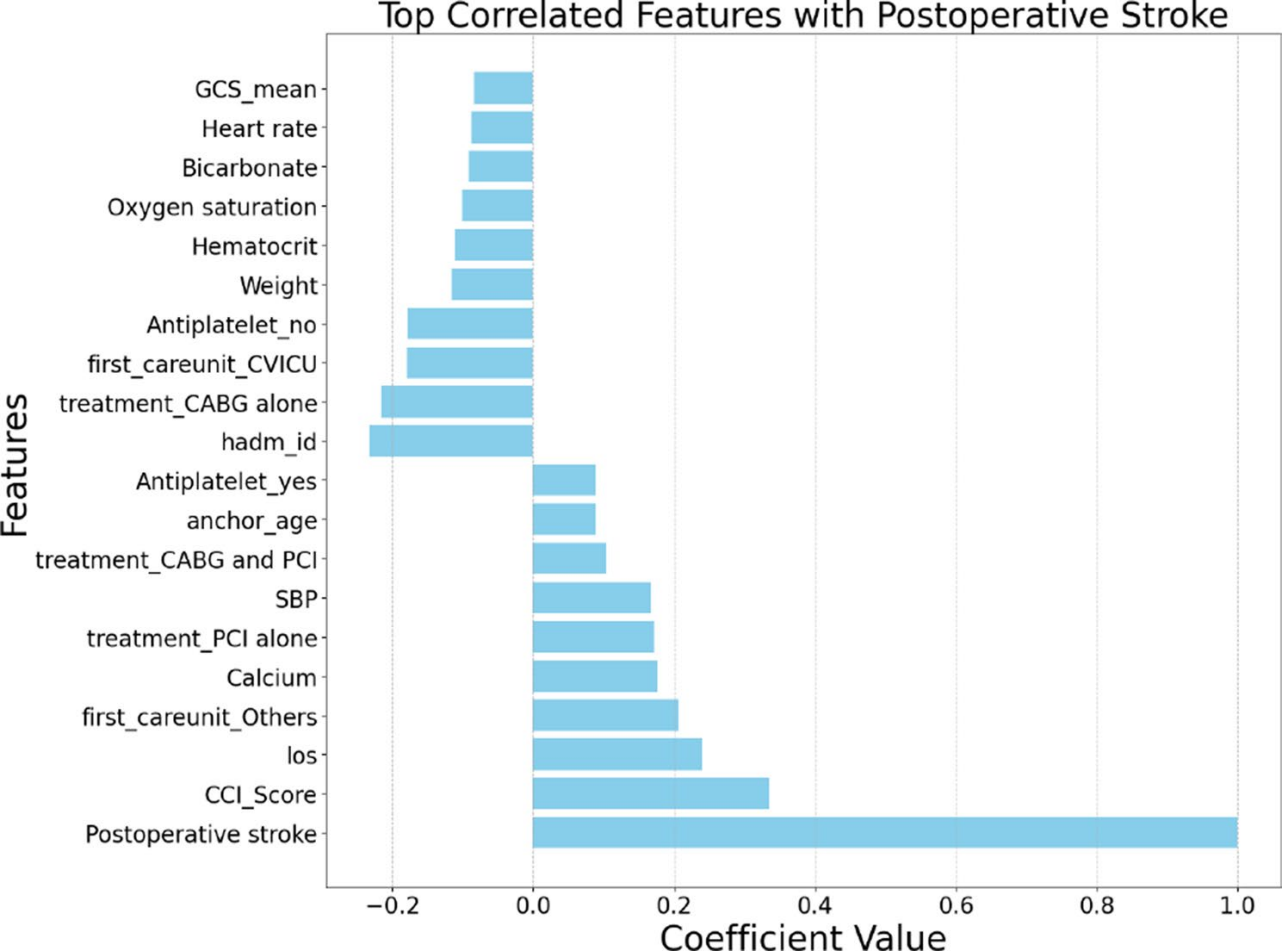
Feature ranking from correlation analysis

**Fig. 3:**
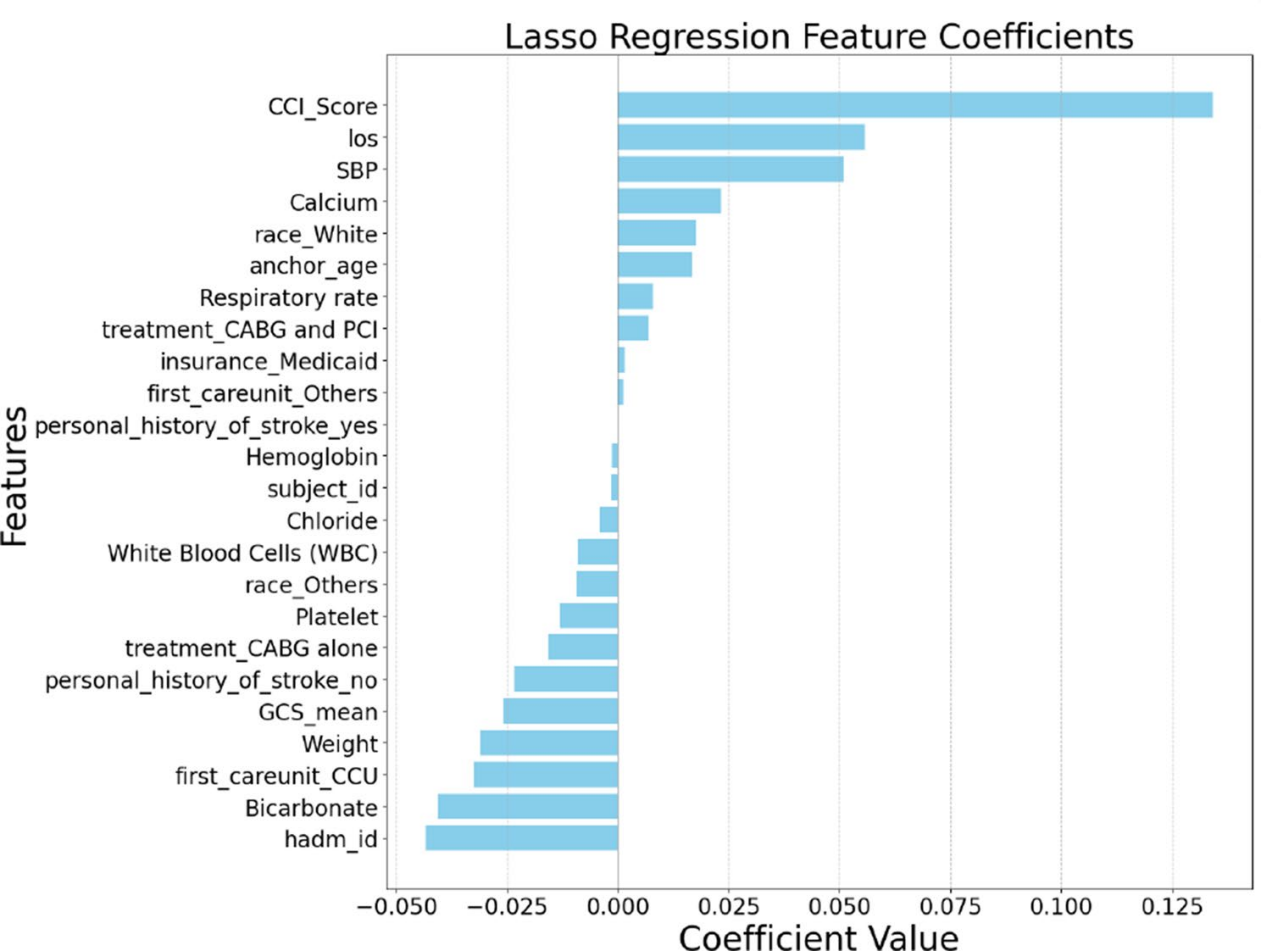
Feature ranking from Lasso regression analysis

**Fig. 4:**
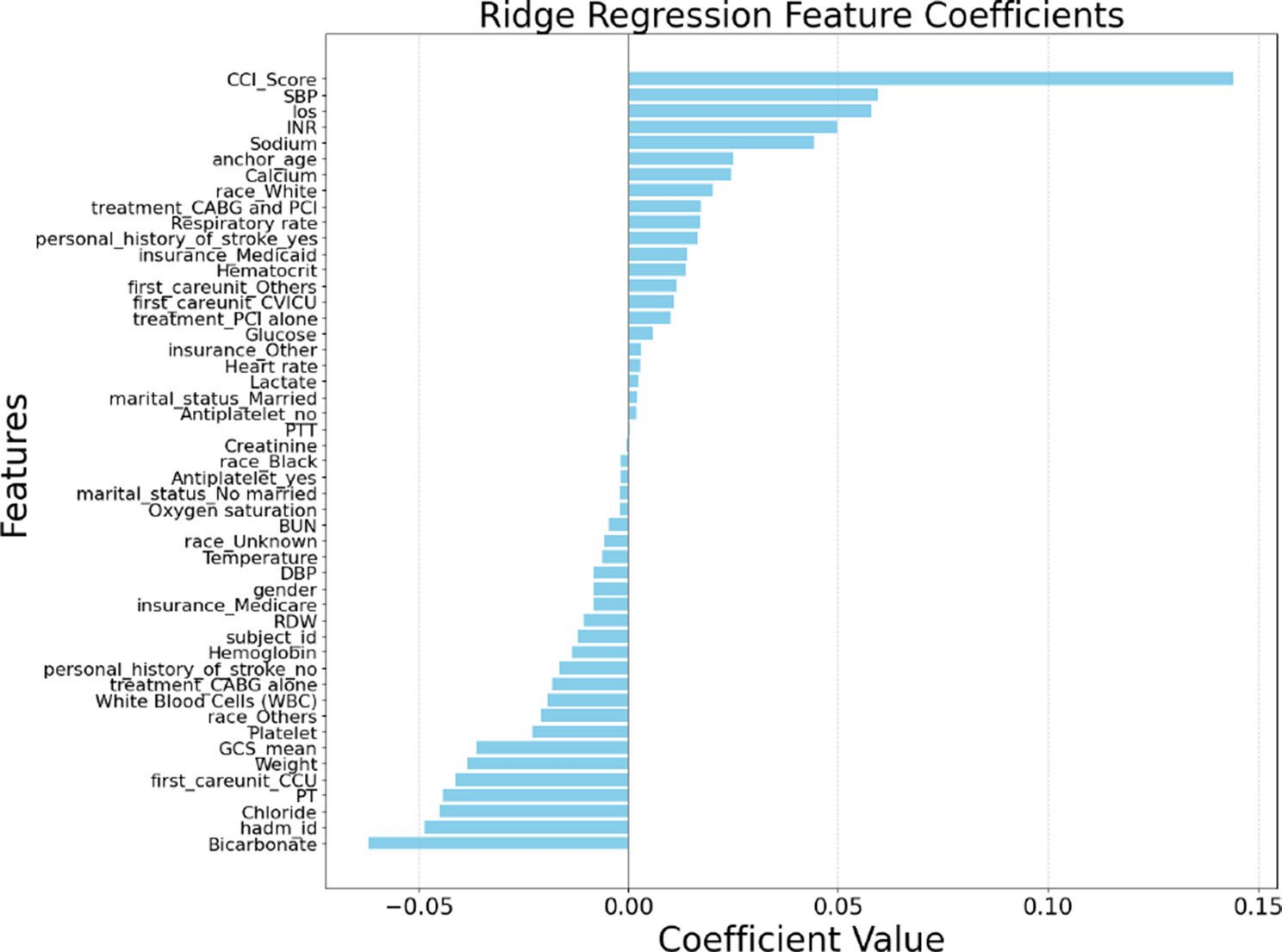
Feature ranking from ridge regression analysis

**Fig. 5:**
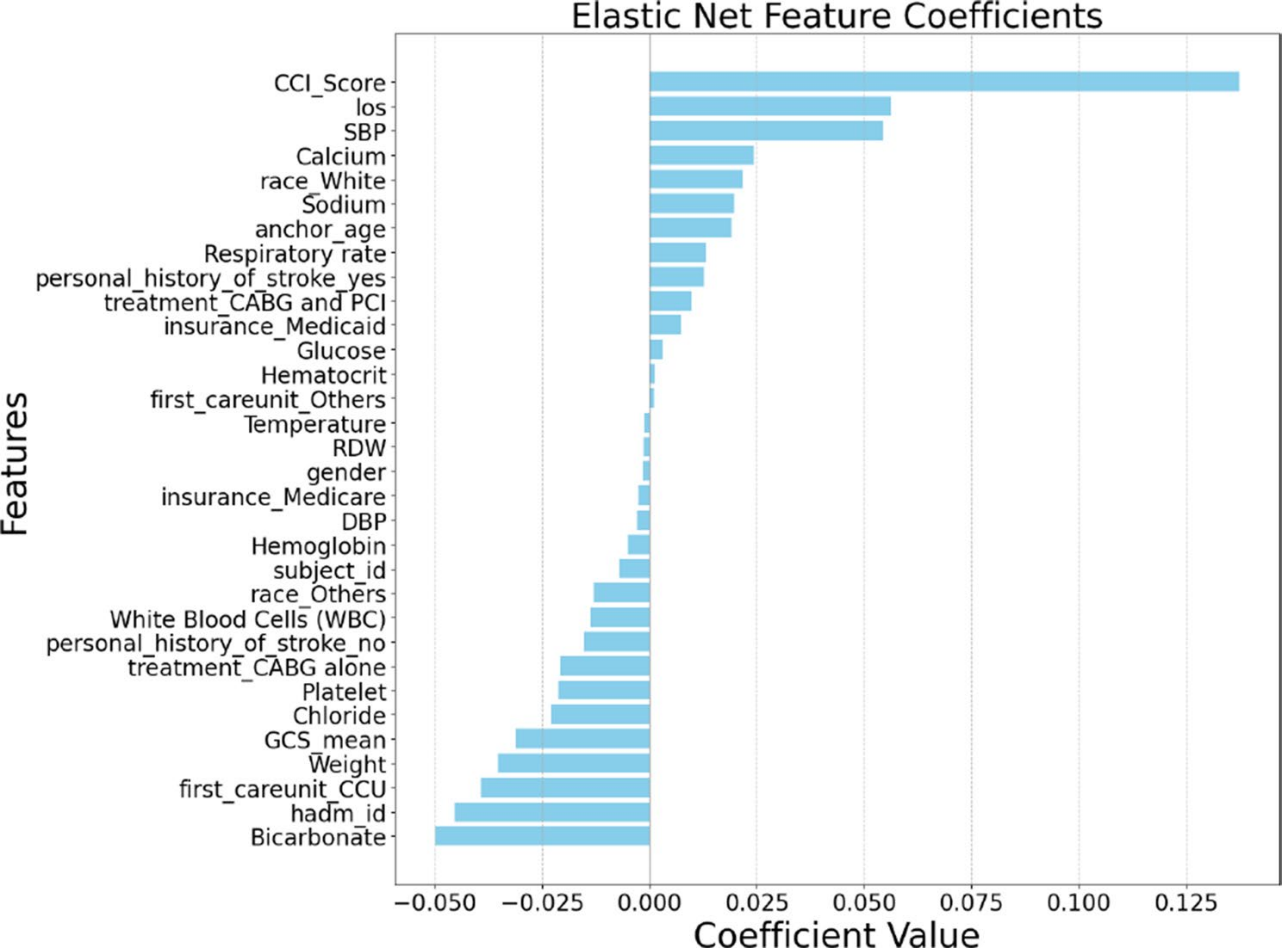
Feature ranking from elastic net feature coefficients analysis

For feature selection, we employed four advanced methodologies: Correlation Analysis, Lasso Regression, Ridge Regression, and Elastic Net. Each technique offered unique insights into feature importance, enabling a robust cross-validation process to identify the most predictive variables. Figs. 2, 3, 4, and 5 depict the feature rankings derived from these methods.

Pearson Correlation Analysis assessed the linear relationship between each feature and postoperative stroke risk, helping identify variables with direct proportional or inverse associations. Lasso Regression, by introducing L1 regularization, not only shrunk coefficient magnitudes but also performed variable selection by setting irrelevant coefficients exactly to zero, promoting sparsity. Ridge Regression, utilizing L2 regularization, retained all features while reducing the impact of multicollinearity, thus capturing subtle yet important predictive contributions. Elastic Net Regression combined both L1 and L2 penalties, balancing variable selection and model stability, particularly beneficial in the presence of correlated predictors common in clinical datasets. For each method, we selected the top ten features based on their ranking and aggregated them through a union operation to form a comprehensive candidate feature set. The final union after the four feature selection processes formed a final set with 14 features.

By leveraging diverse feature selection strategies and emphasizing clinical interpretability, we ensured that the final set of variables was both statistically robust and clinically meaningful for modeling the risk of postoperative stroke in revascularized CAD patients.

### Data preprocessing

Data preprocessing was performed to ensure integrity and enhance input quality for analysis. Missing values were addressed using a dual imputation strategy: Regular Random interpolation was applied for categorical variables, while Random Forest-based interpolation was utilized for numerical variables. This combined approach ensured both consistency and accuracy across the dataset.

To further refine the dataset, Feature Category Reduction was performed to consolidate categories within categorical variables, mitigating the risk of overfitting and enhancing computational efficiency. Imbalances in the dataset, particularly in the target variable (postoperative stroke) and certain feature variables, were addressed using the SMOTE. SOMTE is advantageous due to its simplicity and effectiveness in addressing class imbalance by generating synthetic samples rather than merely replicating existing ones. This enhances the classifier’s generalization ability, reducing the risk of overfitting associated with traditional oversampling methods [36]. SMOTE effectively balanced the dataset by generating synthetic examples of the minority class, ensuring equitable representation and facilitating the detection of rare but clinically significant events. Categorical variables were encoded into numerical formats using label encoding, enabling seamless integration into feature selection models.

The training dataset exhibited a pronounced class imbalance, with significantly fewer postoperative stroke cases compared to non-stroke cases. SMOTE was applied to oversample the minority class, creating synthetic samples to balance the class distribution. This preprocessing step enabled the models to better identify patterns within the minority class, thereby improving predictive performance and ensuring fairness across variables. Importantly, SMOTE was restricted to the training phase only. This strategy enabled the model to learn minority class patterns more effectively during development while preserving the real-world distribution of cases and controls in the validation and test sets. By maintaining natural class imbalance in evaluation datasets, we ensured that model performance metrics reflected realistic clinical deployment conditions and avoided over-optimistic bias.

Through these preprocessing techniques, the dataset was meticulously prepared, enhancing its suitability for feature selection and modeling while improving the ability to detect critical but infrequent outcomes.

### Model training and hyperparameter tuning

The dataset was partitioned into training (70%), validation (15%), and test (15%) sets to facilitate robust model evaluation. Numerical features were standardized to ensure data normalization, critical for scale-sensitive models like SVM and Logistic Regression.

A suite of machine learning models was evaluated for predicting postoperative stroke in a clinical dataset. The models included Logistic Regression (LR), Random Forest (RF), SVM, XGBoost, AdaBoost, Bernoulli Naïve Bayes (NB), and KNN. Each model was optimized using GridSearchCV and cross-validation. For instance, LR achieved its best performance with an L1 penalty and C = 0.001, RF with max_depth = 2 and n_estimators = 15, and SVM with a linear kernel and C = 0.1. Similarly, XGBoost and AdaBoost were optimized for hyperparameters such as maximum depth, learning rate, and ensemble size, while KNN employed dimensionality reduction via Principal Component Analysis (PCA). Specifically, we searched over a range of PCA component values (n_components = 1, 2, 3, 5) using 5-fold cross-validation and selected the optimal number of components based on the highest average ROC AUC score on the validation sets. This approach allowed us to jointly evaluate the best dimensionality-reduction depth and classification performance, while avoiding overfitting. Five principal components were selected for optimal validation performance. Although these models demonstrated strong individual performance, inconsistencies were observed across evaluation metrics.

Among the models, the CatBoost algorithm emerged as the most effective due to its inherent ability to process categorical variables without prior encoding and its robustness when handling highly imbalanced datasets. Comprehensive hyperparameter tuning was performed using GridSearchCV, optimizing parameters such as iterations (5, 10, 20), learning rate (0.5, 0.7, 1.0), depth (1, 2), L2 regularization (500, 1000, 2000), bagging temperature (5, 10, 15), random strength (10, 30, 50), and feature sampling (RSM: (0.1, 0.2, 0.3)).

The final CatBoost model configuration—20 iterations, a learning rate of 1.0, depth of 2, L2 regularization (l2_leaf_reg) of 500, and bagging temperature of 5—achieved superior performance. It recorded the highest cross-validated AUC score of 0.8933 on the training set, with strong generalizability reflected by AUC scores of 0.8486 and 0.8511 on the test and validation sets, respectively. This consistency across datasets highlights the robustness and predictive power of the optimized CatBoost model for identifying postoperative stroke risk in revascularized CAD patients.

All steps of model development—including feature selection, cross-validation, hyperparameter tuning via GridSearchCV, and model training—were strictly confined to the training dataset. Neither the test set nor the validation set was involved in any training or parameter selection to avoid information leakage and to preserve the integrity of performance evaluation.

The validation and test sets were used as independent internal validation cohorts to assess model generalizability and stability across splits. Specifically, the validation set was used to evaluate performance during development for iterative model tuning (e.g., deciding if early stopping or dropout was needed in alternate versions), while the test set served as a completely untouched dataset to provide a final, unbiased estimate of the model’s generalization performance. This three-way split strengthens the credibility of the model’s performance metrics by reducing optimistic bias and improving robustness in internal validation.

### Statistical analysis

We conducted paired t-tests, chi-square tests, and calibration analyses to assess the performance and reliability of the models across training and validation datasets. A threshold p-value of 0.05 was applied to evaluate the statistical significance of differences. The paired t-test results indicated no statistically significant difference between the training and validation AUC scores, confirming the consistency and generalizability of the models. However, chi-square tests revealed a significant difference in feature distributions between the training and validation sets, likely attributable to the SMOTE used to address class imbalance. SMOTE effectively balances datasets by generating synthetic examples for minority classes but can inadvertently alter the original proportions of categorical variables, particularly class labels, thereby impacting the distribution.

Calibration tests were also employed to evaluate the alignment of predicted probabilities with actual event frequencies, a critical aspect of model reliability in clinical applications. Calibration ensures that a model’s predicted probabilities correspond to observed outcomes, enabling actionable and trustworthy risk assessments [37]. While discrimination metrics assess a model’s ability to differentiate between outcomes, poor calibration can undermine the reliability of probability estimates, limiting their practical utility in clinical decision-making.

The calibration analyses of the promoted CatBoost model demonstrated its robustness for clinical applications. Calibration curves showed consistent alignment between predicted probabilities and observed outcomes across both training and validation datasets. This finding indicates that the CatBoost model is well-calibrated, providing reliable probability estimates for postoperative stroke risk. Such calibration reinforces the model’s practical utility, ensuring clinicians can confidently use its outputs for informed decision-making in high-stakes scenarios. These results highlight the importance of combining discrimination and calibration assessments to develop predictive models that are both accurate and clinically actionable.

We considered using DeLong’s test for comparing AUCs between classifiers. However, due to the significant class imbalance in our dataset (only 7% of patients experienced a stroke), DeLong’s test was deemed inappropriate. This test assumes a reasonably balanced number of positive and negative cases and can yield misleading p-values when the minority class is severely underrepresented. In such cases, bootstrap-based AUC confidence intervals and practical differences in performance metrics (e.g., sensitivity and specificity) provide a more robust and informative comparison of models.

### Feature impact

We employed SHAP to evaluate and interpret feature impacts within the predictive model. SHAP is a powerful tool in machine learning, particularly valuable in clinical research, as it provides interpretable and actionable insights into model predictions [38]. By assigning Shapley values to each feature, SHAP quantifies the individual contribution of features to specific predictions, offering a transparent view of the decision-making processes of complex models. This transparency is especially critical in clinical settings, where trust and interpretability are essential for the effective application of predictive models.

In the context of predicting postoperative stroke, SHAP analysis enabled us to uncover the influence of key factors such as patient age, comorbidities, vital signs, and treatment types on the model’s risk assessments. By elucidating these contributions, SHAP fosters confidence in the model’s predictions and supports clinicians in identifying modifiable risk factors, which can inform targeted clinical interventions. Furthermore, SHAP values are instrumental in detecting potential biases or anomalies within the model by highlighting unexpected feature contributions, facilitating necessary adjustments to improve model fairness and accuracy.

In conclusion, SHAP analysis serves as an indispensable component of clinical machine learning by bridging the gap between complex predictive models and the imperative for interpretability in medical decision-making. Through detailed decomposition of feature contributions, SHAP empowers clinicians to make data-driven, informed decisions, ultimately enhancing patient outcomes and fostering trust in AI-driven healthcare solutions.

## Results

### Cohort characteristics

Conducting t-tests for continuous variables and chi-square tests for categorical variables is critical to verify that the training, test, and validation datasets are statistically comparable. This step prevents the introduction of sampling bias, enhances the clinical generalizability of the model, and ensures that the model’s performance can be reliably trusted in real-world patient care. The tables below summarize the statistical test results comparing the distributions of key variables between the training and test sets, and between the training and validation sets. No variables demonstrated statistically significant differences across these splits, as indicated by non-significant p-values in both the t-tests and chi-square tests. This finding confirms that the random splitting strategy preserved the representativeness and balance of the original cohort across all subsets.

Table 1 compares the distributions of 14 selected numerical features between the training and test datasets using two-sample t-tests. The non-significant p-values across all variables indicate that the feature distributions are statistically similar across these two datasets, suggesting a successful random split that preserves clinical comparability.

**Table 1:** Comparison of feature distributions between training and test sets (T-Test results)

Feature	Train Mean	Test Mean	P-value
INR	1.297927	1.265553	0.051865
Anchor Age	67.476049	66.944444	0.192887
Race	2.530405	2.493056	0.262152
LOS	4.860642	5.085432	0.283870
Sodium	138.011685	137.694109	0.310726
Respiratory Rate	18.310966	18.160012	0.403670
Personal History of Stroke	0.008935	0.006944	0.532955
Hematocrit	36.913975	36.787756	0.554946
Treatment	0.580789	0.597222	0.622137
Calcium	5.990564	5.948384	0.765111
Insurance	1.484239	1.488486	0.845985
CCI Score	0.934475	0.922454	0.871829
SBP	112.935802	112.896019	0.889147
Glucose	143.373871	143.723194	0.919798

Table 2 presents a similar comparison of numerical feature distributions, this time between the training and validation sets. As with Table 1, no significant differences were observed, reinforcing the representativeness and consistency of these datasets for model evaluation.

**Table 2:** Comparison of feature distributions between training and validation sets (T-Test results)

Feature	Train Mean	Validation Mean	P-value
Race	2.530405	2.585648	0.070834
Treatment	0.580789	0.629630	0.146487
INR	1.297927	1.322915	0.201860
Sodium	138.011685	137.641644	0.225518
Anchor Age	67.476049	67.076389	0.329857
Glucose	143.373871	140.307801	0.361554
CCI Score	0.934475	1.041667	0.378380
Respiratory Rate	18.310966	18.166024	0.410459
LOS	4.860642	4.999548	0.415841
Hematocrit	36.913975	37.053251	0.528877
Personal History of Stroke	0.008935	0.006944	0.532955
SBP	112.935802	112.771524	0.549440
Insurance	1.484239	1.475694	0.691618
Calcium	5.990564	5.946909	0.758567

Table 3 reports chi-square test results for categorical features between the training and test sets. Again, the p-values suggest no statistically significant differences, validating the balance in categorical distributions achieved during data partitioning.

**Table 3:** Comparison of categorical feature distributions between training and test sets (chi-square test results)

Variable	Chi-Square Statistic	P-value	Degrees of Freedom
Insurance	2.648689	0.265977	2
Race	2.397236	0.494149	3
Treatment	1.233587	0.539672	2
Personal History of Stroke	0.138681	0.709595	1

Table 4 displays chi-square comparisons of categorical features between the training and validation sets. These results confirm that the training and validation cohorts share equivalent distributions for key categorical predictors, ensuring robustness of model generalization and fair evaluation.

**Table 4:** Comparison of categorical feature distributions between training and validation sets (chi-square test results)

Variable	Chi-Square Statistic	P-value	Degrees of Freedom
Treatment	2.178216	0.336517	2
Race	3.151819	0.368805	3
Insurance	1.779094	0.410842	2
Personal History of Stroke	0.138681	0.709595	1

From a clinical perspective, maintaining comparable feature distributions between datasets is essential to ensure that the model learns and is evaluated on patient populations that accurately reflect real-world clinical diversity. It mitigates the risk that the model would overfit to an unrepresentative subset and underperform when applied to new patients. By statistically validating the integrity of the dataset partitions, we strengthened the model’s fairness, transparency, and potential applicability for prospective clinical deployment.

### Evaluation results

The performance of each predictive model was rigorously evaluated using a comprehensive suite of metrics, including the AUC, Receiver Operating Characteristic (ROC) AUC, Precision-Recall Curve, Sensitivity, Specificity, Average Precision, F1 Score, Confusion Matrix, and Log-Loss. This multifaceted evaluation framework provided a holistic understanding of each model’s ability to accurately predict postoperative stroke which is shown in Table 5.

**Table 5:** Performance metrics of models built

Model	AUC	95% CI	Accuracy	Sensitivity	Specificity
**Train**					
Logistic Regression	0.8375	0.8280–0.8457	0.7647	0.8784	0.6510
Random Forest	0.8687	0.8610–0.8766	0.8103	0.8950	0.7255
Support Vector Machines	0.8424	0.8327–0.8500	0.7673	0.7295	0.8050
XGBoost	0.8647	0.8563–0.8723	0.8077	0.9644	0.6510
AdaBoost	0.8570	0.8436–0.8643	0.8103	0.9695	0.6510
Bernoulli Naïve Bayes	0.8074	0.7981–0.8162	0.7282	0.7196	0.7367
K-Nearest Neighbors	0.7551	0.7446–0.7653	0.6905	0.7938	0.5873
CatBoost	0.8933	0.8867–0.9001	0.8123	0.9130	0.7116
**Test**					
Logistic Regression	0.8159	0.7678–0.8576	0.6887	0.9091	0.6704
Random Forest	0.8365	0.7978–0.8724	0.7442	0.8333	0.7368
Support Vector Machines	0.8208	0.7670–0.8641	0.7986	0.7273	0.8045
XGBoost	0.8389	0.7954–0.8731	0.6887	0.9091	0.6704
AdaBoost	0.8198	0.7732–0.8626	0.6887	0.9091	0.6704
Bernoulli Naïve Bayes	0.8254	0.7871–0.8629	0.7604	0.7727	0.7594
K-Nearest Neighbors	0.6666	0.6089–0.7180	0.5891	0.7273	0.5777
CatBoost	0.8486	0.8124–0.8797	0.7419	0.8788	0.7306
**Validation**					
Logistic Regression	0.8532	0.8233–0.8802	0.6782	0.9861	0.6503
Random Forest	0.8305	0.7972–0.8624	0.7257	0.8889	0.7109
Support Vector Machines	0.8441	0.8031–0.8775	0.7847	0.6806	0.7942
XGBoost	0.8263	0.7934–0.8568	0.6782	0.9861	0.6503
AdaBoost	0.8508	0.8215–0.8766	0.6782	0.9861	0.6503
Bernoulli Naïve Bayes	0.8120	0.7699–0.8480	0.7245	0.8194	0.7159
K-Nearest Neighbors	0.6367	0.5691–0.6983	0.5475	0.6528	0.5379
CatBoost	0.8511	0.8203–0.8793	0.7164	0.8472	0.7045

**Fig. 6:**
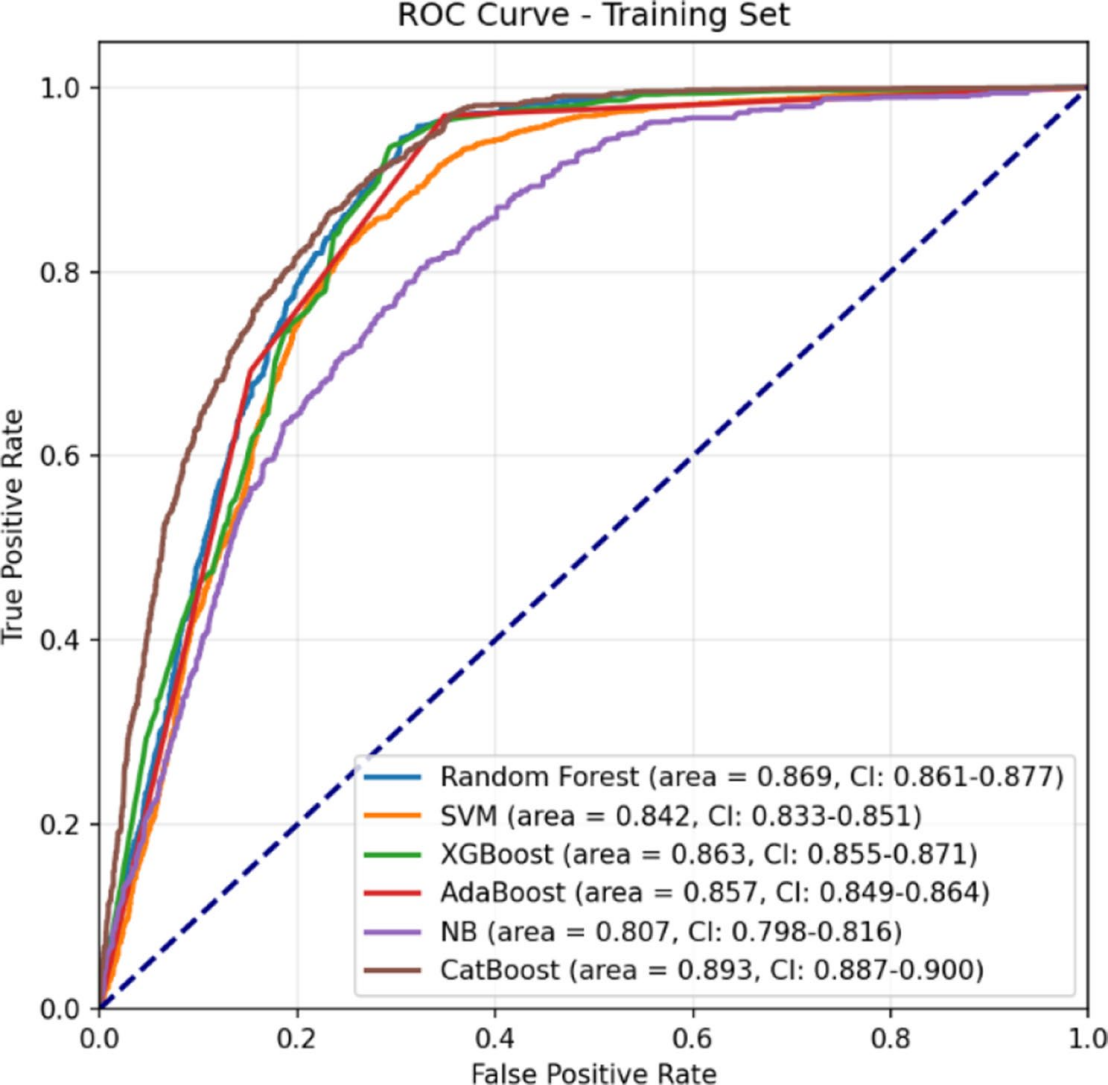
ROC Curve (Train set)

**Fig. 7:**
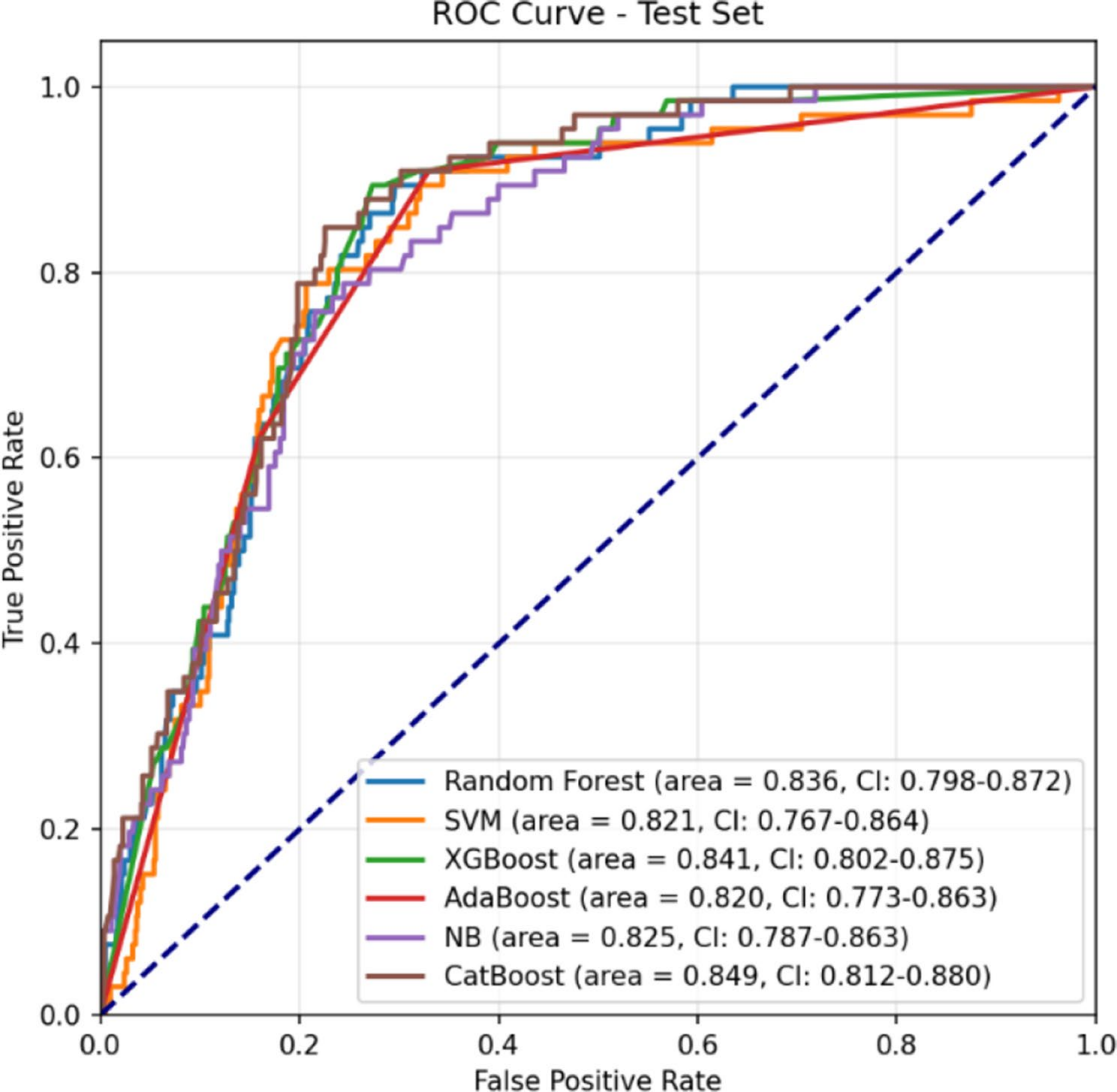
ROC Curve (Test set)

**Fig. 8:**
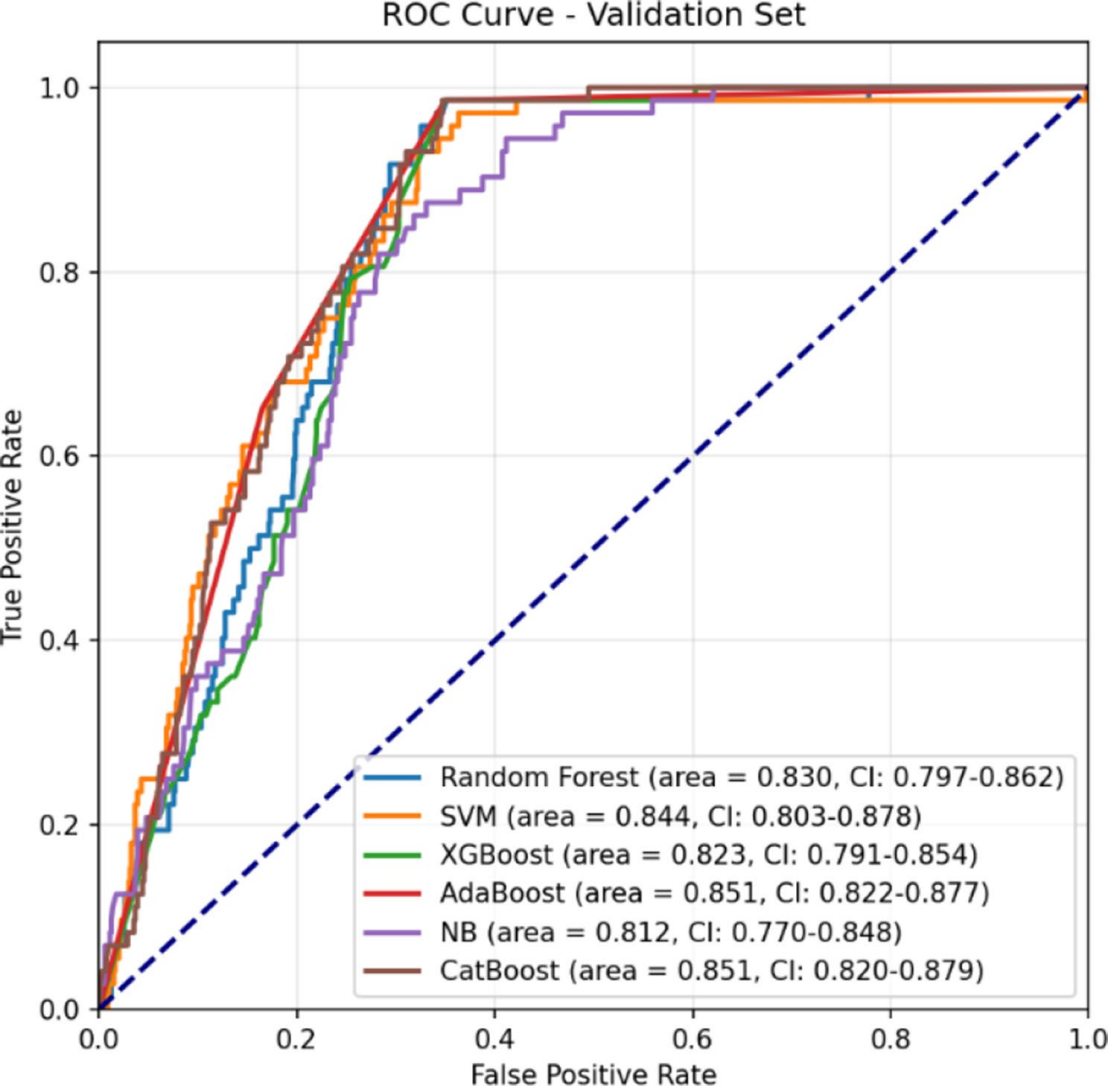
ROC Curve (Validation set)

Among the models, the CatBoost algorithm consistently outperformed its counterparts, achieving the highest AUC scores across all datasets: 0.8486 on the test set and 0.8511 on the validation set. These results underscore CatBoost’s superior capability to discriminate between positive and negative classes. Furthermore, the model demonstrated a balanced performance with a sensitivity of 87.88% and a specificity of 73.06%, effectively minimizing false positive and false negative rates. The ROC curves for all models on the training, test, and validation sets are presented in Figs. 6, 7, and 8, respectively.

**Fig. 9:**
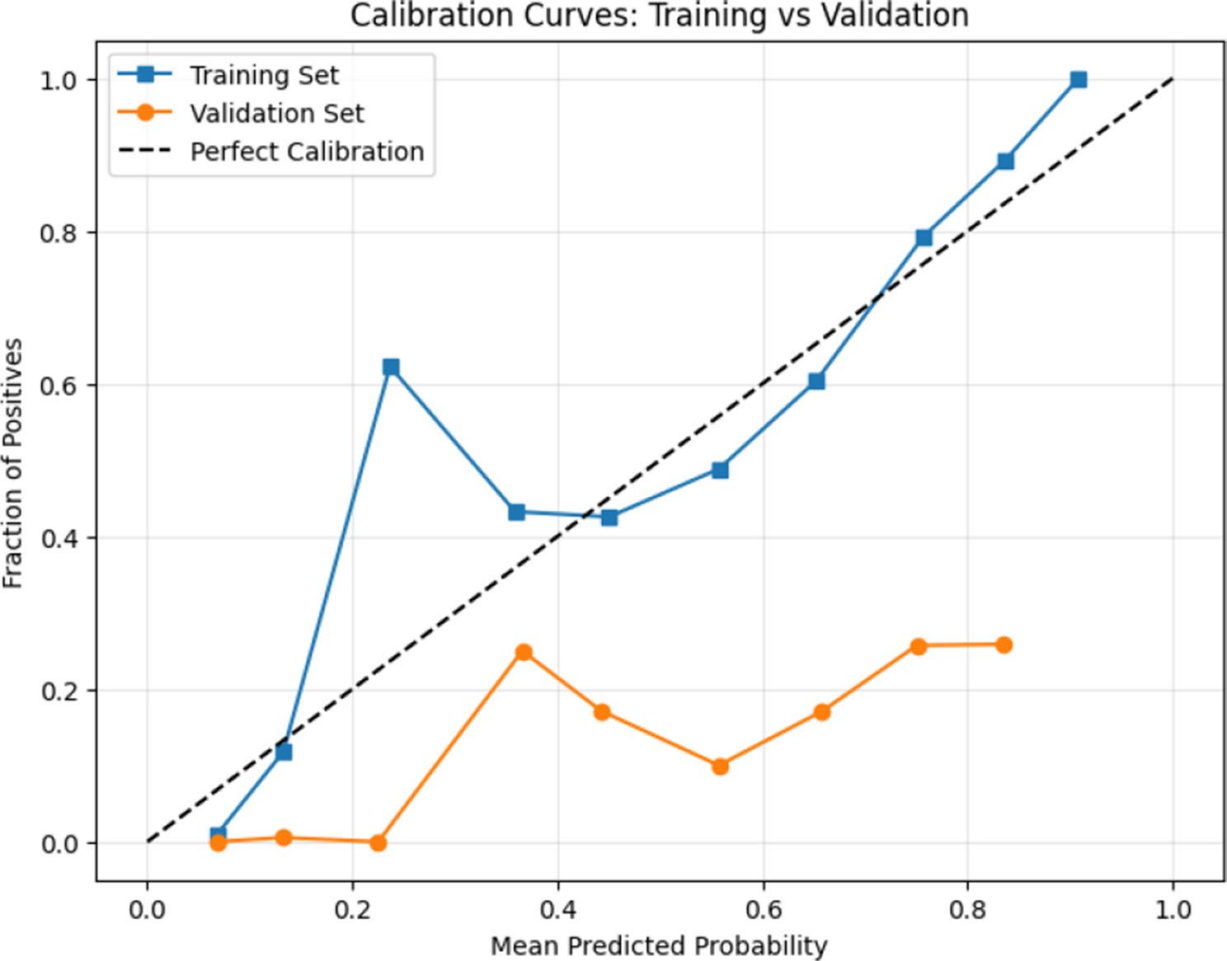
Calibration curves: training vs validation

Calibration testing in Fig. 9 further validated CatBoost’s reliability in probabilistic predictions. The model achieved a Brier score loss of 0.130 on the training set and 0.160 on the validation set, as shown in the calibration curves, the model’s predicted probabilities align reasonably with observed outcomes, though minor deviations are evident. While slight discrepancies in calibration between training and validation sets were observed, these differences are expected due to the inherent variability of validation data and remained within clinically acceptable ranges.

Although Random Forest and XGBoost models delivered competitive performance, CatBoost distinguished itself by seamlessly handling categorical data, maintaining consistent stability across datasets, and offering superior interpretability through SHAP analysis. The model’s ability to directly process categorical features without additional preprocessing steps further highlights its utility in clinical datasets.

In summary, the CatBoost model excelled not only in discriminatory power, as evidenced by its high AUC scores, but also in generating well-calibrated probability estimates. These findings underscore CatBoost’s potential for clinical deployment, where accurate, interpretable, and reliable predictions are vital for risk stratification and informed decision-making.

### SHAP result

**Fig. 10:**
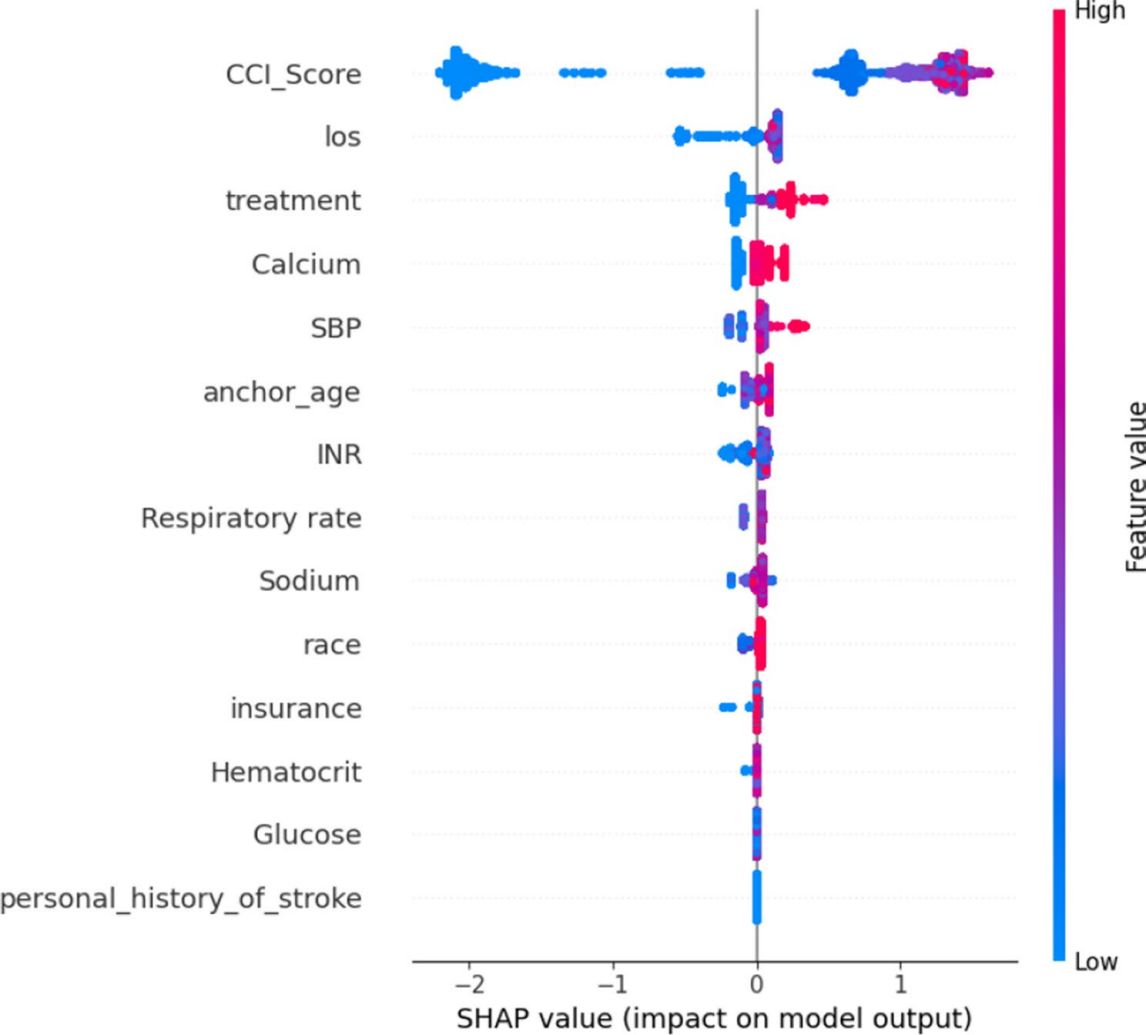
SHAP analysis of top features

To enhance model interpretability, we employed SHAP analysis, which quantifies the contribution of each feature to individual predictions while offering both global and local interpretability. Fig. 10 summarizes the global impact of each feature, ranking variables by their mean absolute SHAP values. The CCI score was identified as the most influential predictor, followed by LOS and treatment type. Notably, higher CCI scores and longer LOS were associated with an increased risk of postoperative stroke, as indicated by positive SHAP values predominantly in high-value regions (red). Conversely, certain treatment types and lower levels of calcium, respiratory rate, and sodium exhibited negative SHAP values, suggesting a protective association.

Beyond feature importance, SHAP analysis provided critical insights into the directionality of feature influence. Positive SHAP values indicated that higher feature values increased predicted stroke risk, while negative SHAP values reflected a protective effect. This directional interpretation enables clinicians to distinguish between modifiable and non-modifiable risk factors, offering actionable intelligence for individualized patient management.

Furthermore, SHAP supports local interpretability at the individual patient level by decomposing a single prediction into feature-specific contributions. Patient-specific SHAP explanations could guide personalized risk communication, targeted interventions, and perioperative planning. Such approaches have been effectively demonstrated in previous studies, including Zhao et al., where SHAP analysis was used to dissect the individual effects of variables on thrombolysis outcomes in STEMI patients [26], and Wang et al., who successfully applied patient-level SHAP interpretation to predict in-hospital mortality in women with ST-segment elevation myocardial infarction [39]. Inspired by these prior works, future research will incorporate individualized SHAP visualizations to further enhance clinical applicability and model transparency in postoperative stroke risk prediction.

## Discussion

This study addresses a critical clinical challenge: predicting the risk of postoperative stroke in patients with CAD undergoing revascularization. Accurate early risk stratification in this population is essential for tailoring perioperative management, optimizing resource allocation, and improving long-term neurological outcomes.

Building upon prior work, we applied a comprehensive data preprocessing framework that integrated both traditional statistical techniques and advanced machine learning interpretability methods. Feature selection was rigorously conducted through Pearson correlation analysis, LASSO, Ridge, and Elastic Net regularization, followed by SHAP interpretability confirmation. This hybrid selection ensured that the final feature set retained only clinically meaningful and statistically robust predictors, balancing model performance with practical usability.

Clinically, several key predictors identified by our model—including the CCI, LOS, INR, and insurance type—align with known stroke risk factors. High CCI scores reflect greater systemic disease burden, which exacerbates vascular vulnerability. Elevated INR values point toward impaired coagulation profiles, heightening stroke susceptibility. Additionally, the association of socioeconomic indicators such as insurance status highlights broader determinants of healthcare access and quality, reinforcing the need for holistic risk management beyond biological metrics.

Compared to traditional statistical models such as logistic regression, which primarily capture linear relationships, our CatBoost model may provide improved capacity to model complex, nonlinear interactions among clinical features. Logistic regression, while historically valuable for risk scoring systems, often underperforms in highly heterogeneous clinical datasets where feature interdependencies are intricate. Our CatBoost-based approach achieved an AUC of 0.8486 on the test set and 0.8511 on the validation set, suggesting improved performance compared to traditional methods in both discrimination and calibration. However, due to differences in cohort characteristics and modeling strategies, these comparisons should be interpreted with caution.

Furthermore, in direct comparison to the previous machine learning-based study by Lulu Li et al., which remains the only known research that specifically targets postoperative stroke prediction in CAD patients undergoing coronary revascularization, our approach demonstrated a substantial performance improvement. While Li et al.’s CatBoost model achieved a test AUC of 0.760, accuracy of 0.664, sensitivity of 0.718, and specificity of 0.660, our optimized CatBoost model achieved a higher AUC of 0.8486, accuracy of 0.7419, sensitivity of 0.8788, and specificity of 0.7306 which is shown in Table 6. Several methodological advancements possibly contributed to this notable improvement. First, we implemented a more rigorous and hybrid feature selection framework that integrated both statistical methods and expert validation, significantly reducing feature redundancy and enhancing generalizability. Second, we addressed class imbalance more effectively by applying SMOTE within a cross-validation framework, minimizing model bias toward the majority class—an aspect that was only partially handled in the prior study. Lastly, our incorporation of SHAP analysis enabled comprehensive interpretability at both the global and patient-specific levels, directly addressing a major limitation of the previous model, which lacked transparency in clinical application. Together, these enhancements result in a model that is not only more accurate but also more reliable and clinically actionable.

**Table 6:** Comparison of CatBoost Model performance between prior and current studies

Metric	Prior Study (Li et al.)	Current Study (Yong et al.)
AUC (Test Set)	0.760	0.8486
Accuracy (Test Set)	0.664	0.7419
Sensitivity (Test Set)	0.718	0.8788
Specificity (Test Set)	0.660	0.7306

Moreover, careful calibration analysis, often overlooked in earlier studies, was rigorously performed, ensuring that predicted stroke risks accurately reflected real-world probabilities. This is particularly crucial in clinical deployment where risk thresholds drive actionable decisions such as intensifying monitoring or initiating early interventions.

Importantly, careful handling of class imbalance using SMOTE within a cross-validation framework helped mitigate overfitting and enhanced model sensitivity to rare stroke events without sacrificing specificity. This balance is crucial in clinical applications, where false negatives can lead to catastrophic missed diagnoses.

## Limitation

Despite these advancements, our study has certain limitations. The exclusive use of the MIMIC-IV database constrains external validation, limiting the applicability of our findings to broader populations. While LOS improves model performance retrospectively, it limits prospective applicability. Future models should consider substituting LOS with early intra-hospital indicators for real-time prediction. Future research should incorporate external datasets from diverse institutions to ensure the generalizability of the model. Additionally, while SMOTE effectively addressed class imbalances, it may alter the original class distribution, requiring careful interpretation of results. Formal statistical comparison of AUCs between models using DeLong’s test was not feasible due to the highly imbalanced class distribution, limiting our ability to assign statistical significance to performance differences. While the Brier scores for the training (0.130) and validation (0.160) sets appeared numerically close, no formal statistical test was performed to assess the significance of this difference. Moreover, visual inspection of the calibration plot reveals substantial calibration drift between the training and validation sets. The model demonstrates good calibration on the training data, with predicted probabilities closely aligning with observed event rates. However, in the validation set, the predicted probabilities systematically overestimate the actual fraction of positives across most probability bins, particularly in the lower probability range. This indicates calibration degradation during validation, suggesting a degree of model overfitting. Future work should consider calibration techniques such as Platt scaling, isotonic regression, or recalibration using a held-out dataset to improve generalization. Finally, translating ML models into routine clinical practice necessitates further exploration of user-friendliness, clinician acceptance, and integration into existing healthcare workflows.

## Conclusion

This study presents a clinically relevant and interpretable machine learning model to predict stroke risk in patients undergoing coronary revascularization. Through a rigorous hybrid feature selection process combining four statistical methods and expert validation, we identified 14 final predictors. Our optimized CatBoost model achieved a test AUC of 0.8486, sensitivity of 0.8788, specificity of 0.7306, and accuracy of 0.7419. Compared to the prior study by Li et al., which reported a test AUC of 0.760, our model demonstrated a notable 9% improvement in discrimination. This performance gain is attributed to advanced feature engineering, cross-validated SMOTE for class balance, and model interpretability via SHAP. These improvements enhance the model’s applicability in real-world settings, offering both high discriminative power and transparency essential for clinical trust. Overall, our findings support the feasibility of using perioperative EHR data to proactively assess stroke risk, potentially guiding timely interventions to improve patient outcomes.

Clinically, this model holds potential as a practical, explainable decision-support tool to identify high-risk patients, enabling targeted preventive interventions, optimized resource deployment, and ultimately, improved neurological and cardiovascular outcomes. Future research should focus on dynamic risk modeling, prospective clinical trials, and deployment in real-world critical care workflows to maximize translational impact.

## Data Availability

The dataset analyzed during the current study is publicly available through the MIMIC-IV database (https://physionet.org/content/mimiciv/2.2/) after completion of the required training and data use agreement.

## Abbreviations

CAD: Coronary Artery Disease
PCI: Percutaneous Coronary Intervention
CABG: Coronary Artery Bypass Grafting
ICU: Intensive Care Unit
LOS: Length of Stay
CCI: Charlson Comorbidity Index
ML: Machine Learning
AUC: Area Under the Curve
SHAP: Shapley Additive Explanations
SMOTE: Synthetic Minority Oversampling Technique
